# Association Between Low-Density Lipoprotein Cholesterol Levels and Risk for Sepsis Among Patients Admitted to the Hospital With Infection

**DOI:** 10.1001/jamanetworkopen.2018.7223

**Published:** 2019-01-18

**Authors:** QiPing Feng, Wei-Qi Wei, Sandip Chaugai, Barbara G. Carranza Leon, Jonathan D. Mosley, Daniel A. Carranza Leon, Lan Jiang, Andrea Ihegword, Christian M. Shaffer, MacRae F. Linton, Cecilia P. Chung, C. Michael Stein

**Affiliations:** 1 Division of Clinical Pharmacology, Department of Medicine, Vanderbilt University Medical Center, Nashville, Tennessee; 2 Department of Biomedical Informatics, Vanderbilt University Medical Center, Nashville, Tennessee; 3 Division of Diabetes, Endocrinology, and Metabolism, Department of Medicine, Vanderbilt University Medical Center, Nashville, Tennessee; 4 Division of Cardiovascular Medicine, Department of Medicine, Vanderbilt University Medical Center, Nashville, Tennessee; 5 Department of Pharmacology, Vanderbilt University, Nashville, Tennessee; 6 Division of Rheumatology and Immunology, Department of Medicine, Vanderbilt University Medical Center, Nashville, Tennessee

## Abstract

**Importance:**

Whether low levels of low-density lipoprotein cholesterol (LDL-C) are associated with increased risk of sepsis and poorer outcomes is unknown.

**Objective:**

To examine the association between LDL-C levels and risk of sepsis among patients admitted to the hospital with infection.

**Design, Setting, and Participants:**

Cohort study in which deidentified electronic health records were used to define a cohort of patients admitted to Vanderbilt University Medical Center, Nashville, Tennessee, with infection. Patients were white adults, had a code indicating infection from the *International Classification of Diseases, Ninth Revision, Clinical Modification*, and received an antibiotic within 1 day of hospital admission (N = 61 502). Data were collected from January 1, 1993, through December 31, 2017, and analyzed from January 24 through October 31, 2018.

**Interventions:**

Clinically measured LDL-C levels (excluding measurements <1 year before hospital admission and those associated with acute illness) and a genetic risk score (GRS).

**Main Outcomes and Measures:**

The primary outcome was sepsis; secondary outcomes included admission to an intensive care unit (ICU) and in-hospital death.

**Results:**

Among the 3961 patients with clinically measured LDL-C levels (57.8% women; mean [SD] age, 64.1 [15.9] years) and the 7804 with a GRS for LDL-C (54.0% men; mean [SD] age, 59.8 [15.2] years), lower measured LDL-C levels were significantly associated with increased risk of sepsis (odds ratio [OR], 0.86; 95% CI, 0.79-0.94; *P* = .001) and ICU admission (OR, 0.85; 95% CI, 0.76-0.96; *P* = .008), but not in-hospital mortality (OR, 0.80; 95% CI, 0.63-1.00; *P* = .06); however, none of these associations were statistically significant after adjustment for age, sex, and comorbidity variables (OR for risk of sepsis, 0.96 [95% CI, 0.88-1.06]; OR for ICU admission, 0.94 [95% CI, 0.83-1.06]; OR for in-hospital death, 0.97 [95% CI, 0.76-1.22]; *P* > .05 for all). The LDL-C GRS correlated with measured LDL-C levels (*r* = 0.24; *P* < 2.2 × 10^−16^) but was not significantly associated with any of the outcomes.

**Conclusions and Relevance:**

Results of this study suggest that lower measured LDL-C levels were significantly associated with increased risk of sepsis and admission to ICU in patients admitted to the hospital with infection; however, this association was due to comorbidities because both clinical models adjusted for confounders, and the genetic model showed no increased risk. Levels of LDL-C do not appear to directly alter the risk of sepsis or poor outcomes in patients hospitalized with infection.

## Key Points

**Question:**

What is the association between low levels of low-density lipoprotein cholesterol and risk of sepsis in patients admitted to the hospital with serious infection?

**Findings:**

In this cohort study of deidentified electronic medical records of patients admitted to the hospital with infection, measured levels of low-density lipoprotein cholesterol in 3961 patients and a low-density lipoprotein cholesterol genetic risk score in 7804 patients were not associated with increased risk of sepsis after adjusting for comorbidities.

**Meaning:**

Levels of low-density lipoprotein cholesterol are not directly associated with the risk of sepsis or poor outcomes in patients hospitalized with infection.

## Teaser

This cohort study of medical records assesses whether low-density lipoprotein cholesterol (LDL-C) levels are associated with an increased risk of sepsis among patients admitted to the hospital with infection.

## Introduction

In the United States, sepsis is a common cause for admission to an intensive care unit (ICU) and contributes to 1 in every 2 to 3 in-hospital deaths.^1,2,3,4^ Sepsis is a complication of infection and is characterized by an uncontrolled systemic inflammatory response, organ failure, poor clinical outcomes, and a high mortality rate. No specific effective treatments are available for sepsis; thus, interest in new approaches to prevent sepsis and treat patients is great.

The effect of lipoproteins on sepsis and its outcomes is 1 such area of interest. Lipoproteins, including low-density lipoproteins (LDL), bind toxic bacterial products such as lipopolysaccharide (LPS) that mediate many of the manifestations of sepsis such as vasodilation, increased capillary permeability, and decreased peripheral vascular resistance.^5,6^ Studies that manipulated LDL levels in animals^5,6^ suggested that LDL protected against LPS-induced mortality.

Studies in humans also suggest that LDL is protective against sepsis but are less clear. Patients with low levels of LDL cholesterol (LDL-C) have an increased risk of sepsis and worse outcomes^7,8,9^; however, these studies do not answer the question whether LDL-C modifies the risk of sepsis and poor outcomes directly, or if it does so indirectly through the effects of comorbid illness. A study performed in community-dwelling adults^10^ showed that low LDL-C levels at entry to the cohort were associated with an increased risk of future sepsis, suggesting that LDL-C levels could affect the risk of sepsis directly.

Understanding the link between LDL-C levels and sepsis is important because newer medications to lower lipid levels (ie, proprotein convertase subtilisin/kexin type 9 [PCSK9] inhibitors) can reduce LDL-C concentrations to very low levels.^11,12^ Also, administering lipoproteins to patients at risk for sepsis is a therapeutic strategy that could potentially prevent or ameliorate the disease. Thus, we tested the hypothesis that low LDL-C levels are directly associated with increased risk of sepsis and poorer outcomes using an epidemiologic approach with careful attention to confounders and a genetic approach that is less prone to confounding. We used a deidentified electronic health record (EHR) repository linked to a DNA biobank to define baseline measured LDL-C levels and an LDL-C genetic risk score (GRS). To define the association between LDL-C and sepsis, we examined the association between measured LDL-C levels as well as the LDL-C GRS and the following 3 outcomes in patients admitted to the hospital with infection: (1) sepsis, (2) admission to an ICU, and (3) in-hospital mortality.

## Methods

### Data Sources

Data were obtained from the Synthetic Derivative, a database that contains a deidentified copy of the EHR for every patient in the Vanderbilt University Medical Center (VUMC) system (>2.5 million patients).^13,14,15^ We used codes from the *International Classification of Diseases, Ninth Revision, Clinical Modification *(*ICD-9-CM*) and *International Statistical Classification of Diseases and Related Health Problems, Tenth Revision *(*ICD-10*) for outcomes and *ICD-9-CM* codes for cohort construction and covariates. The study was approved by the institutional review board of the VUMC, which waived the need for informed consent for the use of deidentified data. This study followed the Strengthening the Reporting of Observational Studies in Epidemiology (STROBE) reporting guideline.

### Cohort Identification

**Figure 1.**
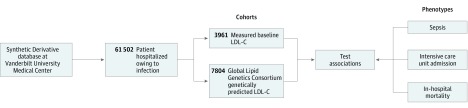
Overview of Clinical and Genetic Approaches LDL-C indicates low-density lipoprotein cholesterol.

We identified white adult patients (≥18 years) who had been admitted to VUMC with an infection from January 1, 1993, through December 31, 2017 (Figure 1 and eFigure in the Supplement). The day of hospital admission was designated day 0. Infection was defined as a billing code indicating infection (eTable 1 in the Supplement) and receipt of an antibiotic within 1 day of hospital admission (ie, on days −1, 0, or +1). We selected *ICD-9-CM* codes for infection based on the criteria of Angus et al^16^ and Donnelly et al,^17^ excluding viral, mycobacterial, fungal, and spirochetal infections. The qualifying antibiotics are shown in eTable 2 in the Supplement. If more than 1 qualifying episode of infection was present in a patient’s EHR, only the first was included; among patients with measured LDL-C levels, the first episode of infection occurring after a qualifying LDL-C measurement was included. We further identified the following 2 subcohorts:

1. Baseline LDL-C levels were measured to reduce the confounding effects of illness. We excluded individuals with evidence of severe chronic illness in the year before the index hospital admission (eFigure in the Supplement). We excluded individuals with conditions such as HIV infection, chronic kidney disease, and liver disease, as well as those who had received chemotherapy for cancer (eTable 3 in the Supplement). We defined the baseline LDL-C level for each individual as the median value of qualifying measurements. Qualifying LDL-C measurements were those performed more than 1 year before the index hospital admission and excluded those performed (1) in hospital; (2) at younger than 18 years; (3) within 30 days of a serum albumin level measurement of less than 3 g/dL (to convert to grams per liter, multiply by 10); and (4) after the first mention of statin therapy in the electronic health record.
2. We identified patients in whom genome-wide genotyping had been performed (including the Infinium Multi-Ethnic Genotyping Array [Illumina], Axiom Biobank Array [Affymetrix], OMNI-Quad [Illumina], HumanOmni5-Quad [Illumina], 660W-Quad [Illumina], and 1M [Illumina]).

### Outcomes

**Figure 2.**
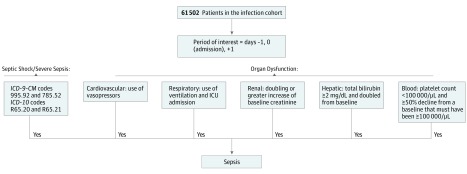
Algorithm to Identify Sepsis Within the Infection Cohort *ICD-9-CM* indicates *International Classification of Diseases, Ninth Revision, Clinical Modification*; *ICD-10*, *International Statistical Classification of Diseases and Related Health Problems, Tenth Revision*; and ICU, intensive care unit. To convert bilirubin to micromoles per liter, multiply by 17.104.

The primary outcome was sepsis, using the Sepsis-3 classification^17^ and a definition of sepsis as concurrent infection and organ dysfunction identified using a validated algorithm to detect sepsis in the EHR with minor modifications (Figure 2 and eMethods in the Supplement).^1^ The algorithm uses a combination of billing codes and clinical criteria and had a sensitivity of 69.7% and specificity of 98.1% in a previous study.^1^ To minimize the contribution of sepsis occurring after surgery or as a complication of events in the hospital, we studied sepsis occurring within 1 day of hospital admission (days −1, 0, and +1). Surveillance studies have shown that most sepsis cases (86.8%) are present on admission to the hospital.^1^ In brief, individuals with infection met the definition of sepsis if they had septic shock or severe sepsis or met any organ dysfunction criterion. Septic shock and severe sepsis were defined by *ICD-9-CM* and *ICD-10* codes that are highly specific (99.3%).^1^ Criteria for organ dysfunction included (1) cardiovascular failure, defined as the use of the vasopressor norepinephrine bitartrate (Levophed) or use of the vasopressors dobutamine hydrochloride or dopamine hydrochloride with a billing code for administration of a vasopressor; (2) respiratory failure, defined as the use of codes for ventilation and admission to an ICU; (3) renal failure, defined as doubling or a greater increase of baseline creatinine level (ie, the lowest creatinine level from 1 year before admission to hospital discharge); (4) hepatic failure, defined as total bilirubin level of 2.0 mg/dL (to convert to micromoles per liter, multiply by 17.104) and doubled from baseline (ie, the lowest total bilirubin occurring from 1 year before admission to hospital discharge); (5) hematologic failure, defined as a platelet count of less than 100 × 10^3^/μL (to convert to 10^9^ per liter, multiply by 1.0) and at least a 50% decline from a baseline that must have been at least 100× 10^3^/μL (ie, the highest platelet count occurring from 1 year before admission to hospital discharge) (see eTable 4 in the Supplement for codes to identify organ failure). Secondary outcomes included admission to the ICU and in-hospital mortality.

### Covariates

We calculated median values for all body mass index (BMI), high-density lipoprotein cholesterol (HDL-C), and triglyceride values in the EHR for each individual; age was ascertained at the time of the index hospital admission. We also established the presence or absence of the comorbidities that constitute the Charlson/Deyo comorbidity index, with modifications.^18,19,20^ For each patient, relevant diagnostic codes in the year before the index hospital admission were grouped (eTable 5 in the Supplement) into PheCodes^20^ using the PheWAS package.^21^ The PheCodes were further grouped into the 16 modified Charlson/Deyo comorbidity categories; the categories of diabetes and diabetes with complications were combined into a single diabetes variable. Four of the comorbidity categories (mild liver disease, moderate and severe liver disease, AIDS/HIV, and metastatic solid tumor) were not present in any individual in the subcohort in whom baseline LDL-C levels had been measured because of the exclusions applied to that group. Each comorbidity group was included as an independent covariate in the analysis. We manually reviewed a sample of EHRs to confirm phenotyping (eMethods and eTable 6 in the Supplement).

### GRS for LDL-C

We generated a GRS for LDL-C using 81 single-nucleotide polymorphisms independently associated (*P* < 5 × 10^−8^) with LDL-C in the meta-analysis of genome-wide studies performed by the Global Lipids Genetics Consortium (eMethods and eTable 7 in the Supplement).^22^ Specifically, the GRS was calculated for each individual by adding the number of minor alleles (0, 1, or 2) weighted for the effect size (β) of the single-nucleotide polymorphism–LDL-C association. The association between the LDL-C GRS and measured LDL-C levels was validated in an independent cohort of 4313 white patients, none of whom were included in the LDL-C GRS cohort.

### Statistical Analysis

Data were analyzed from January 24 to October 31, 2018. We used logistic regression to test the associations between LDL-C levels (measured values and GRS) and outcomes. We estimated odds ratios (ORs) per 1-SD increase and 95% CIs for the association between baseline LDL-C levels or LDL-C GRS and sepsis outcomes (sepsis, ICU admission, and in-hospital mortality) adjusting for age, sex, and comorbidities in the measured LDL-C analyses and in the GRS analyses for age and sex. Sensitivity analyses in the subcohort with measured LDL-C level included (1) additionally adjusting for HDL-C and triglyceride levels, EHR length, and BMI; (2) excluding individuals (n = 473) who had a hospital admission for infection before a qualifying LDL-C measurement; and (3) imposing no exclusions on the LDL-C measurement and using the value closest to admission and imposing no exclusions for comorbidities (n = 12 334). In secondary analyses, we estimated ORs and 95% CIs for quartiles of LDL-C levels and LDL-C GRS using the highest quartile as reference and adjusting as in the primary analyses. Because we tested 3 outcomes, we considered 2-sided *P* < .0167 as statistically significant.

## Results

A total of 61 502 patients met the definition of infection. A total of 3961 patients had clinically measured LDL-C levels (2288 women [57.8%] and 1673 men [42.2%]; mean [SD] age, 64.1 [15.9] years), and 7804 had a GRS for LDL-C (4214 men [54.0%] vs 3590 [46.0%] women; mean [SD] age, 59.8 [15.2] years) (Table 1). A total of 802 patients were in both cohorts.

**Table 1.** Demographic Characteristics

Characteristic	Measured Baseline LDL-C Value (n = 3961)	Genetically Estimated LDL-C Value (n = 7804)
Sex, No. (%)		
Female	2288 (57.8)	3590 (46.0)
Male	1673 (42.2)	4214 (54.0)
Age, mean (SD), y	64.1 (15.9)	59.8 (15.2)
BMI, mean (SD)	30.3 (7.7)	NA
Lipid panel level, mean (SD), mg/dL		
LDL-C	103.4 (32.6)	NA
Triglycerides	161.3 (108.3)	NA
HDL-C	50.84 (17.1)	NA
Myocardial infarction, No. (%)	518 (13.1)	1225 (15.7)
Congestive heart failure, No. (%)	854 (21.6)	1829 (23.4)
Peripheral vascular disease, No. (%)	349 (8.8)	746 (9.6)
Cerebrovascular disease, No. (%)	742 (18.7)	1342 (17.2)
Dementia, No. (%)	310 (7.8)	180 (2.3)
Chronic pulmonary disease, No. (%)	1069 (27.0)	1987 (25.5)
Rheumatic disease, No. (%)	243 (6.1)	429 (5.5)
Peptic ulcer disease, No. (%)	110 (2.8)	296 (3.8)
Mild liver disease, No. (%)	NA	615 (7.9)
Diabetes, No. (%)	1241 (31.3)	2554 (32.7)
Hemiplegia or paraplegia, No. (%)	63 (1.6)	218 (2.8)
Renal disease, No. (%)	144 (3.6)	647 (8.3)
Any malignant neoplasm, including lymphoma and leukemia, except malignant neoplasm of skin, No. (%)	484 (12.2)	1717 (22.0)
Moderate or severe liver disease, No. (%)	NA	571 (7.3)
Metastatic solid tumor, No. (%)	NA	957 (12.3)
AIDS/HIV, No. (%)	NA	105 (1.3)

Abbreviations: BMI, body mass index (calculated as weight in kilograms divided by height in meters squared); HDL-C, high-density lipoprotein cholesterol; LDL-C, low-density lipoprotein cholesterol; NA, not available (excluded for this cohort).

SI conversion factors: To convert LDL-C and HDL-C to millimoles per liter, multiply by 0.0259; and to convert triglycerides to millimoles per liter, multiply by 0.0113.

### Measured Baseline LDL-C Levels

Among the 3961 patients with qualifying baseline LDL-C measurements (Figure 2), 594 patients developed sepsis, 323 were admitted to the ICU, and 82 died during hospitalization. Mean (SD) follow-up in the EHR was 14.2 (6.3) years. Of the 7804 patients who had undergone genotyping, 2520 developed sepsis, 1194 were admitted to ICU, and 243 died during hospitalization. Compared with the cohort with measured baseline LDL-C levels, they were younger, were more likely to be male, and had more renal, hepatic, cancer, and HIV comorbidities because these conditions were exclusion criteria for the measured LDL-C cohort but not the GRS cohort (Table 1).

### Association of Measured Baseline LDL-C Levels and Outcomes

The LDL-C levels were significantly and inversely associated with a risk of sepsis (OR, 0.86; 95% CI 0.79-0.94; *P* = .001) and ICU admission (OR, 0.85; 95% CI, 0.76-0.96; *P* = .008) but not in-hospital mortality (OR, 0.80; 95% CI, 0.63-1.00; *P* = .06). These associations were not significant after adjustment for age, sex, and comorbidity variables (OR for sepsis, 0.96 [95% CI, 0.88-1.06; *P* = .42]; OR for ICU admission, 0.94 [95% CI, 0.83-1.06; *P* = .32]; OR for in-hospital death, 0.97 [95% CI, 0.76-1.22; *P* = .79]) (Table 2). Sensitivity analyses that additionally adjusted for HDL-C and triglyceride levels, EHR length, and BMI yielded similar results (OR for sepsis, 0.95 [95% CI, 0.86-1.04; *P* = .25]; OR for ICU admission, 0.97 [95% CI, 0.86-1.10; *P* = .61]; OR for in-hospital death, 0.98 [95% CI, 0.76-1.24; *P* = .85]). An analysis that excluded individuals with a previous admission to hospital for infection (OR for sepsis, 0.95 [95% CI, 0.86-1.05; *P* = .34]; OR for ICU, 0.97 [95% CI, 0.85-1.11; *P* = .69]; OR for in-hospital death, 1.01 [95% CI, 0.78-1.29; *P* = .95]) was consistent with the main analysis (eTable 8 in the Supplement). Similarly, the sensitivity analysis that used the LDL-C value closest to admission and had no exclusions for comorbidities yielded results similar to those of the primary analysis (OR for sepsis with full adjustment, 0.98 [95% CI, 0.94-1.02; *P* = .24]; OR for ICU admission with full adjustment, 1.04 [95% CI, 0.98-1.09; *P* = .19]; OR for in-hospital death with full adjustment, 0.92 [95% CI, 0.83-1.01; *P* = .08]) (eTable 9 in the Supplement).

**Table 2.** Associations Between LDL-C Level and Sepsis-Related Adverse Outcomes

Cohort	Phenotypes	Unadjusted		Adjusted	
		OR (95% CI)	*P* Value	OR (95% CI)	*P* Value
Measured baseline LDL-C (n = 3961)	Sepsis	0.86 (0.79-0.94)	.001	0.96 (0.88-1.06)^a^	.42^a^
	ICU admission	0.85 (0.76-0.96)	.008	0.94 (0.83-1.06)^a^	.32^a^
	In-hospital death	0.80 (0.63-1.00)	.06	0.97 (0.76-1.22)^a^	.79^a^
GLGC-based LDL-C GRS (n = 7804)	Sepsis	1.02 (0.97-1.07)	.41	1.02 (0.97-1.07)^b^	.37^b^
	ICU admission	1.01 (0.95-1.07)	.84	1.01 (0.95-1.07)^b^	.78^b^
	In-hospital death	0.92 (0.81-1.04)	.17	0.92 (0.81-1.05)^b^	.22^b^

Abbreviations: GLGC, Global Lipid Genetics Consortium; GRS, genetic risk score; ICU, intensive care unit; LDL-C, low-density lipoprotein cholesterol; OR, odds ratio.

^a^ Adjusted for age, sex, and comorbidity covariates.

^b^ Adjusted for age and sex.

### Association of Global Lipids Genetics Consortium–Based LDL-C GRS and Outcomes

The GRS was significantly associated with LDL-C levels (*r* = 0.24; *P* < 2.2 × 10^−16^), accounted for 5.8% of the variability in LDL-C, and was associated with hyperlipidemia (OR, 1.18; 95% CI, 1.15-1.22; *P* = 1.4 × 10^−34^) and coronary atherosclerosis (OR, 1.07; 95% CI, 1.04-1.10; *P* = 4.3 × 10^−7^). However, the GRS was not significantly associated with sepsis, ICU admission, or in-hospital mortality (Table 2) without adjustment (OR for sepsis, 1.02 [95% CI, 0.97-1.07; *P* = .41]; OR for ICU admission, 1.01 [95% CI, 0.95-1.07; *P* = .84]; OR for in-hospital death, OR, 0.92 [95% CI, 0.81-1.04; *P* = .17]) and with adjustment for age and sex (OR for sepsis, 1.02 [95% CI, 0.97-1.07; *P* = .37]; OR for ICU admission, 1.01 [95% CI, 0.95-1.07; *P* = .78]; OR for in-hospital death, 0.92 [95% CI, 0.81-1.05; *P* = .22]).

**Figure 3.**
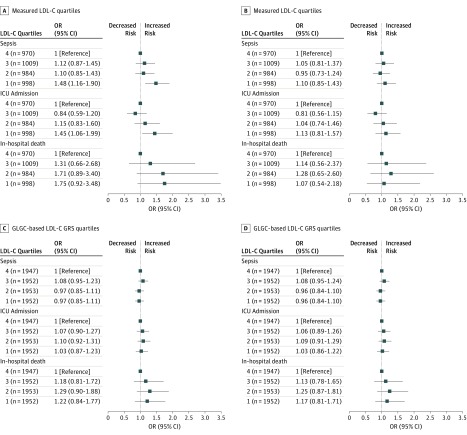
Association Between Low-Density Lipoprotein Cholesterol (LDL-C) Quartiles and Sepsis, Intensive Care Unit (ICU) Admission, and Death The associations between sepsis and its adverse outcomes and measured LDL-C quartiles (top) and genetic risk score (GRS) quartiles (bottom) are shown. Analyses of measured LDL-C quartiles were adjusted for age, sex, and comorbidity variables; analyses of GRS quartiles, for age and sex. GLCG indicates Global Lipids Genetics Consortium; OR, odds ratio.

We also compared the risk of sepsis, ICU admission, and in-hospital mortality within different quartiles for measured LDL-C levels and the GRS. Patients in the lowest quartile of measured LDL-C concentrations had a higher risk of sepsis (OR, 1.48; 95% CI, 0.1.16-1.90) and ICU admission (OR, 1.45; 95% CI, 1.06-1.99) compared with those in the highest quartile of measured LDL-C levels; however, after adjustment for age, sex, and comorbidity variables, none of measured LDL-C level quartiles differed significantly (Figure 3A and B). The lowest LDL-C GRS quartile did not have different risks of sepsis, ICU admission, or in-hospital death compared with the highest quartile (Figure 3C and D).

## Discussion

We used epidemiologic and genetic approaches to define the association between LDL-C levels and sepsis and found that lower LDL-C levels measured in patients at least 1 year before they were admitted to the hospital with infection were associated with increased risk of sepsis, ICU admission, and in-hospital mortality. However, when comorbidities were considered, no association between LDL-C levels and sepsis or its outcomes occurred. Concordant with this finding, genetic risk factors for LDL-C were not associated with sepsis or its outcomes.

Sepsis is a life-threatening and costly condition, which is difficult to anticipate and treat. No specific treatment for sepsis other than early antibiotic therapy and supportive care is available. Strategies to determine which patients with infection are likely to develop sepsis are important because these patients can be targeted for early intensive monitoring. Also, new therapies to prevent or treat sepsis are an important but elusive goal. Thus, LDL-C levels represent an interesting candidate for prevention and treatment of sepsis.

In vitro studies and studies in animals showed that LDL binds toxic bacterial products such as LPS and has other immunologic effects that prevent or ameliorate many of the manifestations of sepsis.^5,6^ In murine models, high endogenous LDL levels (induced by deleting the LDL receptor) protected against the lethal effects of LPS and gram-negative infection.^5^ In contrast, mice that were rendered hypolipidemic had increased LPS-induced mortality that could be reversed by administering exogenous lipoproteins that increased serum lipid levels back to within the physiologic range.^6^ Despite the animal studies suggesting that LDL could affect the outcomes of sepsis directly, few studies have defined this association in patients.

Low LDL-C levels were associated with increased risk of death in patients with community-acquired pneumonia^9^ and with increased risk of sepsis in hospitalized patients^7^; also, sepsis outcomes were worse than those of patients with high LDL-C levels.^8,10^ However, these studies do not necessarily implicate LDL-C directly as a modifier of sepsis because the confounding effects of comorbid illness could account for the findings (ie, sicker patients could have lower LDL-C levels and worse outcomes). An epidemiologic study of approximately 30 000 community-dwelling adults was less prone to confounding and showed that lower LDL-C levels at entry to the cohort were associated with increased risk of future sepsis.^10^ This finding suggested that LDL levels could affect the risk of sepsis directly; however, individuals in the lowest LDL-C quartile were more likely to have preexisting comorbidities, and the possibility of residual confounding remained a concern.^10^

To define the association between LDL-C levels and sepsis more clearly, we took 2 approaches to minimize the confounding effects of comorbid illness. The first used an epidemiologic approach that accounted for comorbidities, and the second used a GRS that should be independent of the confounding effects of illness. Lower measured LDL-C levels were indeed associated with higher risk of sepsis and its complications, although we took steps to exclude LDL-C levels obtained when patients were acutely ill and excluded patients with several comorbid illnesses associated with low LDL-C levels and increased risk of infection. Results of this unadjusted analysis were concordant with those of previous reports.^8,23^ However, no association between LDL-C level and risk of sepsis was found after statistical adjustment that included the comorbidity variables.

The study design sought to reduce the confounding effects of preceding and concomitant illness and thus only included LDL-C measurements obtained more than 1 year before the index admission and excluded individuals with many comorbidities that could lower LDL-C levels and increase the risk of infection. However, this design could have introduced bias and reduced the generalizability of the findings. Two findings suggest that the findings were robust. First, the GRS, which does not anchor on LDL-C measurement and thus provides a way of assessing the validity of the measured LDL-C approach, yielded concordant findings. Second, the sensitivity analysis that imposed no time exclusion on the LDL-C and used the value closest to admission and imposed no exclusions for comorbidities yielded results (eTable 9 in the Supplement) very similar to those of the primary analysis, showing a strong association between low LDL-C levels and risk of sepsis in the unadjusted analysis and a marked attenuation after adjustment for baseline comorbidities. Our finding suggests that although LDL-C levels are indeed associated with increased risk of sepsis, this association is not direct but is due to comorbid illness.

Newer agents to lower LDL-C levels, such as PCSK9 inhibitors, can reduce LDL-C levels to extremely low levels. Although lowering of LDL-C levels with PCSK9 inhibitors has been shown to reduce cardiovascular events in statin-treated patients with coronary artery disease and acute coronary syndrome,^24^ little information is available about other effects of long-term low LDL-C levels. Traditional postmarketing drug safety approaches to determine these long-term effects will require years of study. By applying a clinical and genetic approach, we observed no association between low LDL-C levels and sepsis. Therefore, therapy to lower LDL-C levels is unlikely to increase sepsis risk for patients with serious infection.

The genetic approaches further supported the interpretation that LDL-C levels are not directly associated with sepsis. The genetic architecture of circulating lipid levels is well understood; the Global Lipids Genetics Consortium has published a series of large-scale genome-wide association studies,^25,26^ and these observations have been widely used to construct GRSs for lipids levels. A GRS combined with mendelian randomization has been a powerful tool for testing the potential causal relationships between lipid levels and specific diseases. Studies using this genetic approach suggested a direct association between high LDL-C levels and the risk of cardiovascular disease^22^ and the presence of aortic valve calcium and incidence of aortic stenosis^27^; between high triglyceride levels (but not HDL-C levels) and the risk of cardiovascular disease^22^; and between low LDL-C levels and the risk of diabetes.^26,28^ Similarly, an LDL-C GRS approach suggested that low LDL-C levels were not associated with Alzheimer disease, although this had been a clinical association of concern.^29^ In the present study, the Global Lipids Genetics Consortium–based LDL-C GRS was significantly associated with LDL-C levels extracted from the EHR in patients who had had them measured, and with hyperlipidemia and coronary atherosclerosis, but was not significantly associated with the risk of sepsis or its adverse outcomes.

### Strengths and Limitations

The present study has several strengths. Large clinical trials in patients with sepsis are difficult to perform; we were able to use existing clinical and genetic information from a large EHR system linked to a DNA bank that provided longitudinal information, including information about drugs, diagnoses, and laboratory findings in a large number of patients to address the questions of interest. The extensive clinical data available allowed us to restrict the measured LDL-C cohort to individuals who had their LDL-C levels measured at least 1 year before the index admission to hospital. This restriction not only minimized the effect of the current illness on LDL-C levels but also provided clinical diagnoses in the preceding year to adjust for comorbidities. In addition, using a clinical and a genetic approach maximized our ability to distinguish whether any association between LDL-C levels and sepsis was likely to be direct or indirect.

However, this study also has several limitations. First, the EHR provides information about a range of variables that facilitates statistical adjustment; however, we did not perform a randomized clinical study. Thus, results could be influenced by unmeasured confounders. This possibility seems unlikely to be an important consideration because the confounders included accounted for much of the association between LDL-C levels and sepsis. Second, patients could have received care outside of the VUMC system. Thus, covariates could be incomplete, and although we set out to capture the first hospitalization due to infection, a patient could have been treated elsewhere previously for a serious infection that was not captured. Third, although the GRS was significantly associated with measured LDL-C levels, it explained only a small percentage of overall LDL-C variation in the population. This is the case even for large-scale genetic analyses of circulating LDL-C levels^25^; however, the genetic component has been strong enough for the reliable detection of several disease associations.^22,26,27,28^ Nevertheless, those associations were between LDL GRSs and chronic diseases; the association with acute illness may be more difficult to detect. Fourth, the LDL GRS did not capture LDL changes due to environmental factors, such as diet and lifestyle. Fifth, we cannot exclude the possibility of unrelated competing effects of LDL-C levels and comorbidities on sepsis risk, although little evidence supports it. Sixth, we only studied LDL-C levels and the relatively common variants reported to be associated with them in genome-wide association studies. Concentrations of other lipids such as HDL-C^9^ and the presence of other variants in genes such as *PCSK9* that affect LDL-C but were not identified in genome-wide association studies could be important because they may affect the outcomes of sepsis.^23^ Seventh, we studied white patients admitted to a tertiary-level US hospital with infection who had LDL-C levels measured at least 1 year before admission or had undergone genotyping, which limits the generalizability of the findings.

## Conclusions

In this study, lower measured LDL-C levels were significantly associated with increased risk of sepsis and admission to ICU in patients admitted to hospital with infection, but this finding was owing to comorbidities, because adjusted clinical models and genetic models showed no increased risk. Levels of LDL-C do not appear to alter the risk of sepsis or poor outcomes directly in patients hospitalized with infection.

## Notes

Supplement. **eMethods.** Data Sources and Analyses **eReferences** **eFigure.** Outline of Cohort Identification **eTable 1.** List of *ICD-9* Codes to Identify Individuals Hospitalized With Infection **eTable 2.** List of Antibiotics **eTable 3.** Exclusion Codes **eTable 4. **Codes for Identification of Organ Failure **eTable 5.** PheCode Algorithms for Generating Comorbidity Covariates **eTable 6.** Manual Review to Confirm Sepsis Algorithm **eTable 7.** Variants Used to Generate GLGC-Based LDL-C Genetic Risk Score **eTable 8.** Sensitivity Analyses in Subcohort With Measured LDL-C **eTable 9.** Associations Between Closest LDL-C and Sepsis-Related Adverse Outcomes
