# Supplementary material for: Association Between Low-Density Lipoprotein Cholesterol Levels and Risk for Sepsis Among Patients Admitted to the Hospital With Infection
Source: JAMA Netw Open. 2019 Jan 18;2(1):e187223. doi: 10.1001/jamanetworkopen.2018.7223 (PMC6447031; doi:10.1001/jamanetworkopen.2018.7223)
Supplement: Supplement. — eMethods. Data Sources and Analyses eReferences eFigure. Outline of Cohort Identification eTable 1. List of ICD-9 Codes to Identify Individuals Hospitalized With Infection eTable 2. List of Antibiotics eTable 3. Exclusion Codes eTable 4. Codes for Identification of Organ Failure eTable 5. PheCode Algorithms for Generating Comorbidity Covariates eTable 6. Manual Review to Confirm Sepsis Algorithm eTable 7. Variants Used to Generate GLGC-Based LDL-C Genetic Risk Score eTable 8. Sensitivity Analyses in Subcohort With Measured LDL-C eTable 9. Associations Between Closest LDL-C and Sepsis-Related Adverse Outcomes [file jamanetwopen-2-e187223-s001.pdf]

Supplementary Online Content

Feng Q, Wei W-Q, Chaugai S, et al. Association between low-density lipoprotein cholesterol levels and risk for sepsis among patients admitted to the hospital with infection. *JAMA Netw Open*. 2019;2(1):e187223. doi:10.1001/jamanetworkopen.2018.7223

eMethods. Data Sources and Analyses

eReferences

eFigure. Outline of Cohort Identification

eTable 1. List of *ICD-9* Codes to Identify Individuals Hospitalized With Infection

eTable 2. List of Antibiotics

eTable 3. Exclusion Codes

eTable 4. Codes for Identification of Organ Failure

eTable 5. PheCode Algorithms for Generating Comorbidity Covariates

eTable 6. Manual Review to Confirm Sepsis Algorithm

eTable 7. Variants Used to Generate GLGC-Based LDL-C Genetic Risk Score

eTable 8. Sensitivity Analyses in Subcohort With Measured LDL-C

eTable 9. Associations Between Closest LDL-C and Sepsis-Related Adverse Outcomes

This supplementary material has been provided by the authors to give readers additional information about their work.

© 2019 Feng Q et al. *JAMA Network Open.*

eMethods. Data Sources and Analyses

Data sources

Data were obtained from the Synthetic Derivative, a database that contains a de-identified copy of the EHR for every patient in the Vanderbilt University Medical Center (VUMC) system, a large teaching hospital and its affiliated network (N>2.5 million). This de-identified EHR is scrubbed of all Health Insurance Portability Accountability Act (HIPAA) identifiers. It incorporates diagnostic and procedure codes, demographics, clinical notes, patient history, problem lists, laboratory values and medications from which researchers can extract phenotypes such as diagnoses, treatments, and outcomes.[1–3] ICD9 and ICD10 codes were used for outcomes and ICD9 codes for cohort construction and covariates.

EHR-based sepsis algorithm

Sepsis was defined as concurrent infection and organ dysfunction (according to Sepsis-3) occurring within one day of hospital admission (days -1, 0, and +1) using an algorithm to detect sepsis using EHR data[4] with minor modifications. The modifications were as follows: (1) the original algorithm only included ICD9 codes for septic shock; we also included ICD10 codes for septic shock (ICD10 codes R65.20 and R65.21); (2) vasopressor initiation was identified by use of levophed (noprepinephrine bitartrate) or use of dobutamine or dopamine and a billing code for administration of a vasopressor (ICD9-CM procedure code 00.17 or ICD10-PCS Procedure codes 3E033XZ, 3E043XZ, 3E053XZ and 3E063XZ) because dobutamine or dopamine alone had low specificity for identifying patients in whom it was used as a pressor; (3) we did not use serum lactate levels as a criterion because they were seldom available; (4) we excluded individuals who had scheduled cardiothoracic surgery, because the algorithms did not reliably identify the reason for artificial ventilation or ICU admission and some of these patients had an infection and received an antibiotic.

Manual Chart Review

© 2019 Feng Q et al. *JAMA Network Open.*

We manually reviewed EHRs to confirm phenotyping, including (1) 13 with very low LDL-C levels, (2) 4 with an extremely long hospital admission, (3) 6 with very high LDL-C levels, and (4) the performance of the sepsis criteria in 50 EHRs. All patients reviewed met the inclusion and exclusion criteria, outlier variables were not errors, and outcomes criteria performed well (**Supplementary Table 8)**. We also confirmed in-hospital death by manually reviewing the records of all 82 individuals who had baseline LDL-C levels measured and who died during hospitalization.

Genetic risk score for LDL-C

Genotype imputation was conducted on the Michigan Imputation server[5] with minimac3,[23] using the Haplotype Reference Consortium reference panel, version r1.1.[6 7] We generated a GRS for LDL-C using 81 SNPs independently associated ($p<5x10^{-8}$)with LDL-C in the meta-analysis of genome wide studies performed by the Global Lipids Genetics Consortium (GLGC) (**Supplementary Table 5, Supplementary Methods**).[7] For each SNP, the estimated effect size and strength of association were obtained.[8] Genotypes were extracted from imputed genotype data for 7804 Whites. The GRS was calculated for each individual by adding the number of minor alleles (0, 1, or 2) weighted for the effect size (β) of the SNP-LDL association. The association between the LDL GRS and LDL-C levels was validated in an independent cohort of 4313 white subjects who had LDL-C levels measured and had been genotyped. None of them were included in our LDL GRS cohort. To further confirm the validity of LDL-C GRS, we used logistic regression to test associations between the GRS and candidate phecodes, 272.1 (hyperlipidemia) and 411.4 (coronary atherosclerosis), within whites from the Synthetic Derivative.

© 2019 Feng Q et al. *JAMA Network Open.*

eReferences

1. Wei W-Q, Denny JC. Extracting research-quality phenotypes from electronic health records to support precision medicine. *Genome Med*. 2015;7(1):41. doi:10.1186/s13073-015-0166-y

2. Xu H, Stenner SP, Doan S, Johnson KB, Waitman LR, Denny JC. MedEx: a medication information extraction system for clinical narratives. *J Am Med Inf Assoc*. 2010;17:19-24. doi:17/1/19 [pii] 10.1197/jamia.M3378

3. Denny JC, Bastarache L, Ritchie MD, et al. Systematic comparison of phenome-wide association study of electronic medical record data and genome-wide association study data. *Nat Biotechnol*. 2013;31(12):1102-1110. doi:10.1038/nbt.2749

4. Rhee C, Dantes R, Epstein L, et al. Incidence and Trends of Sepsis in US Hospitals Using Clinical vs Claims Data, 2009-2014. *JAMA*. 2017;318(13):1241-1249. doi:10.1001/jama.2017.13836

5. Das S, Forer L, Schönherr S, et al. Next-generation genotype imputation service and methods. *Nat Genet*. 2016;48(10):1284. doi:10.1038/ng.3656

6. Consortium the HR, McCarthy S, Das S, et al. A reference panel of 64,976 haplotypes for genotype imputation. *Nat Genet*. 2016;48(10):1279. doi:10.1038/ng.3643

7. Do R, Willer CJ, Schmidt EM, et al. Common variants associated with plasma triglycerides and risk for coronary artery disease. *Nat Genet*. 2013;45(11):1345-1352. doi:10.1038/ng.2795

8. Global Lipids Genetics Consortium. Discovery and refinement of loci associated with lipid levels. *Nat Genet*. 2013;45(11):1274-1283. doi:10.1038/ng.2797

© 2019 Feng Q et al. *JAMA Network Open.*

eFigure. Outline of Cohort Identification

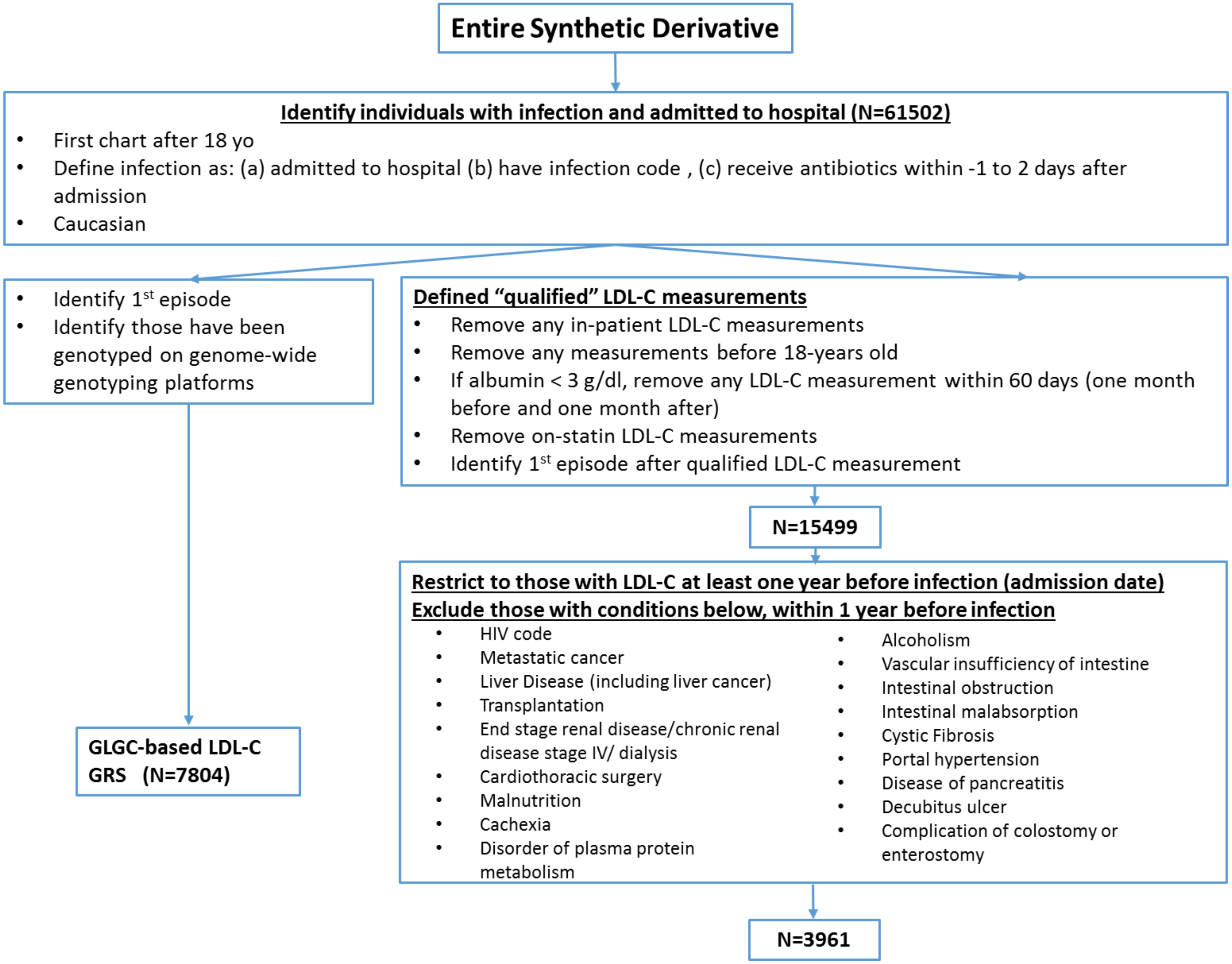

© 2019 Feng Q et al. *JAMA Network Open.*

eTable 1. List of *ICD-9* Codes to Identify Individuals Hospitalized With Infection

001, Cholera	510, Empyema
002, Typhoid/paratyphoid fever	513, Lung/mediastinum abscess
003, Other salmonella infection	540, Acute appendicitis
004, Shigellosis	541, Appendicitis not otherwise specified
008, Intestinal infection not otherwise classified	542, Other appendicitis
009, Ill-defined intestinal infection	562.01, Diverticulitis of small intestine without hemorrhage
023, Brucellosis	562.03, Diverticulitis of small intestine with hemorrhage
027, Other bacterial zoonoses	562.11, Diverticulitis of colon without hemorrhage
032, Diphtheria	562.13, Diverticulitis of colon with hemorrhage
033, Whooping cough	566, Anal and rectal abscess
034, Streptococcal throat/scarlet fever	567, Peritonitis
035, Erysipelas	569.5, Intestinal abscess
036, Meningococcal infection	569.83, Perforation of intestine
038, Septicemia	572.0, Abscess of liver
039, Actinomycotic infections	572.1, Portal pyemia
040, Other bacterial diseases	575.0, Acute cholecystitis
041, Bacterial infection in other diseases not otherwise specified	590, Kidney infection
098, Gonococcal infections	597, Urethritis/urethral syndrome
320, Bacterial meningitis	599.0, Urinary tract infection not otherwise specified
322, Meningitis, unspecified	601, Prostatic inflammation
324, Central nervous system abscess	614, Female pelvic inflammation disease
420, Acute pericarditis	615, Uterine inflammatory disease
421, Acute or subacute endocarditis	616, Other female genital inflammation
461, Acute sinusitis	681, Cellulitis, finger/toe
462, Acute pharyngitis	682, Other cellulitis or abscess
463, Acute tonsillitis	683, Acute lymphadenitis
464, Acute laryngitis/tracheitis	686, Other local skin infection
465, Acute upper respiratory infection of multiple sites/not otherwise specified	711.0, Pyogenic arthritis
481, Pneumococcal pneumonia	730, Osteomyelitis
482, Other bacterial pneumonia	790.7, Bacteremia
485, Bronchopneumonia with organism not otherwise specified	996.6, Infection or inflammation of device/graft
486, Pneumonia, organism not otherwise specified	998.5, Postoperative infection

© 2019 Feng Q et al. *JAMA Network Open.*

491.21, Acute exacerbation of obstructive chronic bronchitis	999.3, Infectious complication of medical care not otherwise classified.
494, Bronchiectasis	
Note: whenever 3- or 4- digits were listed, all sub-codes were included.	

© 2019 Feng Q et al. *JAMA Network Open.*

eTable 2. List of Antibiotics

List of antibiotics

Amikacin	Amoxicillin	cefalexin
Amphotericin B	ceftriaxone	cefadroxil
Ampicillin	tazobactam	tazobactam
Azithromycin	levofloxacin	oxacillin
Bacitracin	doxycycline	dicloxacillin
Capreomycin	ciprofloxacin	nafacillin
Cefuroxime	metronidazole	carbenicillin
Chloramphenicol	sulfamethoxazole	piperacillin
Chlortetracycline	trimethoprim	ticarcillin
Clindamycin	cephalexin	sulbactam
Colistin	tetracycline	clavalanate
Cycloserine	cefuroxime	gatifloxacin
Erythromycin	cefdinir	norfloxacin
fidaxomicin	moxifloxacin	lomefloxacin
Gentamicin Sulfate (USP)	clarithomycin	moxifloxacin
Gramicidin	lincomycin	trovafloxacin
Griseofulvin	dalbavancin	sparfloxacin
Kanamycin	oritavancin	
Natamycin	telavancin	
Neomycin	imipenem	
Netilmicin	meropenem	
Nystatin	doripenem	
Oxytetracycline	cefotaxime	
Paromomycin	cefpodoxime	
Penicillin G	cefixime	
Polymyxin B	ceftazidime	
Rifabutin	ceftizoxime	
Rifampin	cefdinir	
rifapentine	ceftibaten	
rifaximin	cefotetan	

© 2019 Feng Q et al. *JAMA Network Open.*

Streptomycin	cefaclor	
Tetracycline	loracarbet	
Tobramycin	cefepime	
Vancomycin	cefazdin	

© 2019 Feng Q et al. *JAMA Network Open.*

eTable 3. Exclusion Codes

PheCode				ICD9 and CPT codes		
585.32	530.2	577.2		33508	0258T	V58.1
585.1	560.4	70.4		33510	0259T	90780
571.51	573	559		33511	33405	90781
260	509.3	853		33512	33410	96400
585.31	441	270.38		33513	33411	96408
572	260.1	587		33514	33425	96425
571	707.1	263		33516	33426	96520
571.5	557	155		33517	33427	96530
571.8	536.7	270.3		33518	33430	96401-96549
571.81	260.22	573.4		33519	33460	V42.*
317.1	577.1	155.1		33521	33463	
577	573.2			33522	33464	
317	71.1			33523	33465	
260.2	499			33533	33470	
317.11	260.3			33534	33475	
560	573.5			33535	33530	
585.34	270			33536	33641	
				33530	33675	
				33572	33676	
				35600	33677	
				0256T	33681	
				0257T		

© 2019 Feng Q et al. *JAMA Network Open.*

eTable 4. Codes for Identification of Organ Failure

ICD code for administration of a vasopressor	ICD code for ventilation	CPT code for ventilation	CPT code for ICU admission
00.17	96.7	94002	99291
3E033XZ	96.71	94003	99292
3E043XZ	96.72	94004	
3E053XZ	5A1935Z	94656	
3E063XZ	5A1945Z	94657	
	5A1955Z		

© 2019 Feng Q et al. *JAMA Network Open.*

eTable 5. PheCode Algorithms for Generating Comorbidity Covariates

Comorbidities	Deyo's ICD-9-CM	phecode
Myocardial infarction	410.x, 412.x	411.2
Congestive heart failure	428.x	428, 428.1, 428.2, 428.3, 428.4
Peripheral vascular disease	443.9, 441.x, 785.4,	443.9, 442.1, 442.11, 791, 459.7,
	430.x–438.x	430, 430.1, 430.2, 430.3, 433, 433.1, 433.11, 433.12, 433.2, 433.21, 433.3, 433.31, 433.5, 433.6, 433.8,
Dementia	290.x	290, 290.1, 290.13, 290.16
Chronic pulmonary disease	490.x–505.x, 506.4	497, 496.2, 496.21, 496.1, 495, 495.2, 495.1, 495.11, 496.3, 500.1, 500.2, 496, 500
Rheumatic disease	710.0, 710.1, 710.4, 714.0–714.2, 714.81,	709/695.42, 709.3, 709.4, 714, 714.1, 717
Peptic ulcer disease	531.x–534.x	531, 531.1, 531.2, 531.3, 531.4, 531.5
Mild liver disease	571.2, 571.4–571.6	317.11, 70.4, 571.51, 571.6,
Diabetes	250.0–250.7	250, 250.1, 250.2, 250.11, 250.21, 250.15, 250.12, 250.13, 250.22, 250.23, 250.24,250.25,
Hemiplegia or paraplegia	344.1, 342.x	344, 342
Renal disease	582.x, 583–583.7, 585.x, 586.x, 588.x	580.1, 580.11, 580.12, 580.14, 580.3, 580.32, 585.4, 585.34, 585.2, 588 , 588.1, 588.2,
Any malignancy, including lymphoma and leukemia, except malignant neoplasm of skin	140.x–172.x, 174.x.–195.8, 200.x–208.x	145.1-145.5, 145, 149.1-149.3, 149, 150,151,159.2,153.2,153.3,153,155.1,151,159.3,157,159.4,159,164,174.11,174.2,230,182,180,180.1,184.1,184.11,184.2,185,187.2,187,187.1,189.21,189.11,189.12,190,191.11,191.1,193,194
Moderate or severe liver disease	456.0–456.21, 572.2–572.8	454, 530.2, 571.8, 571.81
Metastatic solid tumor	196.x–199.1	198, 198.1, 198.2, 198.3, 198.4, 198.5, 198.6, 198.7 , 195.1
AIDS/HIV	042.x–044.x	71, 71.1

© 2019 Feng Q et al. *JAMA Network Open.*

eTable 6. Manual Review Results to Confirm the Sepsis Algorithm

Supplementary Table. Manual review results to confirm the sepsis algorithm												
	Severe sepsis / Sepsis shock		Cardiovascular failure		Renal failure		Hepatic failure		Hematologic failure		Respiratory failure	
	reviewed	algorithm	reviewed	algorithm	reviewed	algorithm	reviewed	algorithm	reviewed	algorithm	reviewed	algorithm
**1 (yes)**	13	13	18	18	18	17	21	19	21	20	13	13
**0 (no)**	37	35	32	31	32	32	29	29	29	28	37	34

Reviewed = gold standard by reviewing records

Algorithm= number correct according to algorithm

© 2019 Feng Q et al. *JAMA Network Open.*

eTable 7. Variants Used to Generate GLGC-Based LDL-C Genetic Risk Score

rs ID	chr	pos	a1	a2	LDL-C beta
rs10903129	1	25641524	A	G	-0.033
rs1998013	1	55730618	T	C	-0.38
rs4587594	1	62906518	A	G	-0.049
rs6603981	1	92766395	T	C	0.034
rs646776	1	109620053	T	C	0.16
rs1010167	1	110000250	C	G	-0.025
rs267733	1	149225460	A	G	0.033
rs2642438	1	219036651	A	G	-0.035
rs903319	1	219052434	T	C	-0.027
rs2587534	1	232915962	A	G	0.039
rs1367117	2	21117405	A	G	0.12
rs515135	2	21139562	T	C	-0.14
rs1260326	2	27584444	T	C	0.021
rs3817588	2	27584716	T	C	0.026
rs6544713	2	43927385	T	C	0.081
rs4148218	2	43953086	A	G	-0.044
rs2710642	2	63003061	A	G	0.024
rs17508045	2	118293189	T	C	0.049
rs2030746	2	121025958	T	C	0.021
rs16831243	2	135478814	T	C	0.038
rs2287623	2	169538401	A	G	-0.022
rs1250229	2	216012629	T	C	-0.024
rs11563251	2	234344123	T	C	0.035
rs9875338	3	12271469	A	G	-0.027
rs7640978	3	32508014	T	C	-0.039
rs17345563	3	133691893	A	G	0.036
rs7703051	5	74661243	A	C	0.073
rs4530754	5	122883315	A	G	0.028

© 2019 Feng Q et al. *JAMA Network Open.*

rs6882076	5	156322875	T	C	-0.046
rs2294261	6	16217142	A	C	0.033
rs1800562	6	26201120	A	G	-0.062
rs2247056	6	31373469	T	C	-0.025
rs17789218	6	100706818	T	C	0.024
rs868943	6	116444196	A	G	-0.026
rs2297374	6	160495975	T	C	-0.033
rs1564348	6	160498850	T	C	-0.048
rs12670798	7	21573877	T	C	-0.034
rs4722551	7	25958351	T	C	-0.039
rs2073547	7	44548856	A	G	-0.049
rs217386	7	44567220	A	G	-0.036
rs4240624	8	9221641	A	G	0.067
rs10102164	8	55584167	A	G	0.032
rs2326077	8	59548473	T	C	-0.034
rs2737252	8	116733072	A	G	-0.031
rs2980885	8	126543488	A	G	-0.031
rs7832643	8	145094645	T	G	0.034
rs3780181	9	2630759	A	G	0.045
rs1883025	9	106704122	T	C	-0.03
rs8176720	9	135122694	T	C	0.033
rs579459	9	135143989	T	C	-0.067
rs2255141	10	113923876	A	G	0.03
rs10832962	11	18612847	T	C	0.032
rs174532	11	61305450	A	G	0.035
rs1535	11	61354548	A	G	0.053
rs10790162	11	116144314	A	G	0.076
rs11220462	11	125749162	A	G	0.059
rs653178	12	110492139	T	C	0.023
rs6489818	12	110794963	A	G	0.028
rs1186380	12	119860799	T	C	-0.024
rs1169288	12	119901033	A	C	-0.038

© 2019 Feng Q et al. *JAMA Network Open.*

rs4942486	13	31851388	T	C	0.024
rs8017377	14	23953727	A	G	0.03
rs9989419	16	55542640	A	G	0.028
rs2288002	16	70614783	A	G	-0.029
rs2000999	16	70665594	A	G	0.065
rs314253	17	7032374	T	C	0.024
rs4791641	17	8101874	T	C	-0.02
rs7225700	17	42746803	T	C	-0.03
rs6511720	19	11063306	T	G	-0.22
rs688	19	11088602	T	C	0.054
rs10401969	19	19268718	T	C	0.12
rs6859	19	50073874	A	G	0.084
rs7254892	19	50081436	A	G	-0.49
rs492602	19	53898229	A	G	-0.029
rs364585	20	12910718	A	G	-0.025
rs2328223	20	17793921	A	C	-0.03
rs7264396	20	33618155	T	C	-0.025
rs6016381	20	38613850	T	C	0.036
rs6065311	20	39157752	T	C	-0.042
rs1800961	20	42475778	T	C	-0.069
rs5763662	22	28708703	T	C	0.077

Note: rs2954022 was removed due to multiple alleles

- **The table was adopted from reference 25 (***Nat Genet*. 2013;45(11):1345-1352)

© 2019 Feng Q et al. *JAMA Network Open.*

eTable 8. Relationship Between LDL-C Concentrations and Outcomes

Phenotypes	Measured baseline LDL-C, N=3961			
	Unadjusted		adjusted[1]	
	Odds Ratio	P-value	Odds Ratio	P-value
**Sepsis**	0.86 (0.79 - 0.94)	0.00127	0.95 (0.86 - 1.04)	0.25
**ICU admission**	0.85 (0.76 - 0.96)	0.00795	0.97 (0.86 - 1.10)	0.61
**in-hospital death**	0.80 (0.63 - 1.00)	0.0549	0.98 (0.76 - 1.24)	0.85

Phenotypes	Measured baseline LDL-C, excluding those with prior infection, N=3488			
	Unadjusted		adjusted[2]	
	Odds Ratio	P-value	Odds Ratio	P-value
**Sepsis**	0.85 (0.77 - 0.94)	0.00105	0.95 (0.86 - 1.05)	0.34
**ICU admission**	0.86 (0.76 - 0.98)	0.025	0.97 (0.85 - 1.11)	0.69
**in-hospital death**	0.80 (0.62 - 1.02)	0.084	1.01 (0.78 - 1.29)	0.95

adjusted[1] = adjusted for age, sex, comorbidity covariates, BMI, Trigs, HDL and EHR length;
adjusted[2] = adjusted for age, sex and comorbidity covariates

© 2019 Feng Q et al. *JAMA Network Open.*

eTable 9. Associations Between Closest LDL-C and Sepsis-Related Adverse Outcomes

eTable 9. Associations Between Closest LDL-C and Sepsis-Related Adverse Outcomes

		most recent LDL-C, n=12334			
		**Odd Ratio**	**95% CI**		**P-value**
**Sepsis**	**without adj**	0.9069	0.8713	0.9436	1.53E-06
	**adj. age, sex and comorbidity index groups**	0.9758	0.9363	1.0167	0.24
**ICU admission**	**without adj**	0.9695	0.9188	1.0222	0.26
	**adj. age, sex and comorbidity index groups**	1.0367	0.9823	1.0933	0.19
**in-hosptal death**	**without adj**	0.8036	0.7296	0.8833	7.36E-06
	**adj. age, sex and comorbidity index groups**	0.9176	0.8329	1.0082	0.078

© 2019 Feng Q et al. *JAMA Network Open.*
